# Transmission of Bluetongue Virus Serotype 8 by Artificial Insemination with Frozen–Thawed Semen from Naturally Infected Bulls

**DOI:** 10.3390/v13040652

**Published:** 2021-04-09

**Authors:** Kris De Clercq, Leen Vandaele, Tine Vanbinst, Mickaël Riou, Isra Deblauwe, Wendy Wesselingh, Anne Pinard, Mieke Van Eetvelde, Olivier Boulesteix, Bart Leemans, Robert Gélineau, Griet Vercauteren, Sara Van der Heyden, Jean-François Beckers, Claude Saegerman, Donal Sammin, Aart de Kruif, Ilse De Leeuw

**Affiliations:** 1 Unit of Exotic and Particular Diseases, Scientific Directorate Infectious Diseases in Animals, Sciensano, 1180 Brussels, Belgium; ilse.deleeuw@sciensano.be (I.D.L.); 2 Department of Reproduction, Obstetrics and Herd Health, Ghent University, 9820 Merelbeke, Belgium; leen.vandaele@ilvo.vlaanderen.be (L.V.); wendy.wesselingh@anicura.nl (W.W.); mieke.vaneetvelde@ugent.be (M.V.E.); b.leemans@uu.nl (B.L.); Aart.DeKruif@UGent.be (A.d.K.); 3 UE-1277 Plateforme d’Infectiologie Expérimentale (PFIE), Centre de Recherche Val de Loire, Institut National de Recherche Pour l’Agriculture, l’Alimentation et l’Environnement (INRAE), 37380 Nouzilly, France; mickael.riou@inrae.fr (M.R.); anne.pinard@inrae.fr (A.P.); olivier.boulesteix@inrae.fr (O.B.); robert.gelineau0648@orange.fr (R.G.); 4 The Unit of Entomology, Department of Biomedical Sciences, Institute of Tropical Medicine, 2000 Antwerp, Belgium; ideblauwe@itg.be; 5 Department of Pathology, Bacteriology and Poultry Diseases, 9820 Merelbeke, Belgium; griet.vercauteren@zoolyx.be (G.V.); vanderheyden.sara@gmail.com (S.V.d.H.); 6 Département des Sciences Fonctionnelles (DSF), Faculty of Veterinary Medicine, University of Liège, Quartier Vallée 2, 4000 Liège, Belgium; jfbeckers@uliege.be; 7 Research Unit in Epidemiology and Risk Analysis Applied to Veterinary Sciences (UREAR-ULg), Fundamental and Applied Research for Animal and Health (FARAH) Center, Department of Infectious and Parasitic Diseases, Faculty of Veterinary Medicine, University of Liège, 4130 Liege, Belgium; claude.saegerman@uliege.be; 8 Department of Agriculture Food and the Marine Laboratories, Backweston, W23 X3PH Co. Kildare, Ireland; donal.sammin@agriculture.gov.ie

**Keywords:** bluetongue, bluetongue virus, serotype 8, BTV-8, transmission, artificial insemination, semen, cattle

## Abstract

Transmission of bluetongue (BT) virus serotype 8 (BTV-8) via artificial insemination of contaminated frozen semen from naturally infected bulls was investigated in two independent experiments. Healthy, BT negative heifers were hormonally synchronized and artificially inseminated at oestrus. In total, six groups of three heifers received semen from four batches derived from three bulls naturally infected with BTV-8. Each experiment included one control heifer that was not inseminated and that remained BT negative throughout. BTV viraemia and seroconversion were determined in 8 out of 18 inseminated heifers, and BTV was isolated from five of these animals. These eight heifers only displayed mild clinical signs of BT, if any at all, but six of them experienced pregnancy loss between weeks four and eight of gestation, and five of them became BT PCR and antibody positive. The other two infected heifers gave birth at term to two healthy and BT negative calves. The BT viral load varied among the semen batches used and this had a significant impact on the infection rate, the time of onset of viraemia post artificial insemination, and the gestational stage at which pregnancy loss occurred. These results, which confirm unusual features of BTV-8 infection, should not be extrapolated to infection with other BTV strains without thorough evaluation. This study also adds weight to the hypothesis that the re-emergence of BTV-8 in France in 2015 may be attributable to the use of contaminated bovine semen.

## 1. Introduction

Bluetongue virus (BTV) (family Reoviridae, genus *Orbivirus*) is the etiologic agent of bluetongue (BT), a noncontagious, vector-borne disease of domestic and wild ruminants [1]. BT occurs worldwide and is classified by the World Organization for Animal Health (OIE) as a notifiable disease. It mainly affects sheep, while in other ruminants BT is typically asymptomatic, except in white tailed deer [2,3]. Cattle, sheep, white tailed deer and red deer are central to the epidemiology of BTV infection because they can remain viraemic for up to several weeks and serve as reservoir hosts from which biting midges (*Culicoides* spp.) can transmit BTV to other susceptible host animals [3,4,5,6]. BT epizootics can have a severe socioeconomic impact [7], impacting on cattle and sheep farmers through productivity loss, restrictions and/or onerous regulatory requirements for international trade in livestock and germplasm [8]. BTV-8 emerged in West-Central Europe in August 2006, infecting susceptible livestock in the Netherlands, Belgium, Germany, Luxembourg, and Northern France [9,10,11]. The virus continued to circulate in that part of Europe during the following three years (2007–2009), with infection extending in all directions from the initial focus and associated with high morbidity and mortality [12,13,14]. In most of these affected countries, no further cases of BTV-8 were found after the period 2010–2011 thanks to mass vaccination using an inactivated BTV-8 vaccine [15], until BTV-8 re-emerged in France in 2015 [16] and spread to neighbouring countries [17]. Courtejoie et al. (2018) [18] suggested that BTV-8 may have continued to circulate at low levels during the period of 2010–2015. However, comparison of whole viral genome sequences showed that the virus isolated in 2015 was identical to the virus circulating in Europe in 2010 [19,20].

The BTV-8 epizootic that occurred in Europe during the period 2006–2010 had many features that were unusual for bluetongue. In contrast to the disease associated with most BTV strains circulating in Southern Europe before 2006, cattle infected with BTV-8 displayed clinical signs of infection [21,22,23]. BTV-8 was able to cross the placenta in pregnant dams and ewes [24,25,26]. Although transplacental infection with wild-type BTV strains was described before (BTV-4 in sheep [27] and probably BTV-8 in cattle [28]), it remained a very rare phenomenon. The teratogenic consequences of BTV-8 transplacental transmission during the European epizootic included abortion, stillbirth and malformation of the central nervous system (CNS), including hydranencephaly [29,30,31]. Congenital malformation of the CNS in calves and neurological signs were again observed in calves in France in the period 2018–2019 [32]. Another feature of BTV-8 infection was its impact on fertility in bulls and rams with virus shedding in semen [33,34] and a transient impairment of sperm production [35,36]. In previous instances where BTV had been isolated from bovine semen, blood cells (erythrocytes and leucocytes) were also present in semen and were thought to have carried the virus into the semen from the bloodstream. However, semen from bulls that were naturally infected with BTV-8 in Belgium in 2007, and in which the virus was detected, was found not to contain any blood cells [33]. Virus shedding in semen is particularly important because transmission of BTV by artificial insemination (AI) has been demonstrated using semen from experimentally infected bulls [37,38]. These findings were the basis for stringent guidelines being introduced, governing trade in frozen semen between BT-affected and unaffected regions [8]. An increased tropism for placental tissue [39] and semen viral shedding [40] are distinct features of attenuated BTV strains present in modified live BTV vaccines [41]. The potential for BTV transmission to occur by AI with BTV-contaminated bovine semen, and the potential for the virus to survive indefinitely in frozen stocks of contaminated semen, were recently advanced as hypotheses for the re-emergence of BTV-8 in France in 2015 [20].

This feature of BTV-8 regarding its excretion in semen deserves further investigation, especially given the potential for international transmission of BT to occur by artificial insemination. The efficacy of measures to prevent BTV transmission via commercial stocks of semen has been quantified [42], but essential information on viral shedding and transmission were not available. The aim of the present study was to determine the possibility of BTV infection occurring in breeding females that were previously unexposed to BTV, following AI with frozen batches of BTV-contaminated semen collected from bulls that had been naturally infected with BTV-8, and to document any impact on pregnancy and the foetus.

## 2. Materials and Methods

### 2.1. Animals

A total of 20 maiden heifers (24–26 months old) of beef breeds (Simmental, Charolais and crossbreeds of Simmental or Charolais) were sourced in Ireland, which was officially recognised as BTV-free at that time (2010). The heifers were clinically inspected, treated with an insecticide and certified for transport as per regulations. All heifers were screened before transport and before AI for specific antibodies to BTV, bovine viral diarrhoea virus (BVD), infectious bovine rhinotracheitis (IBR), enzootic bovine leucosis (EBL), Neospora and Q fever, and for the presence of viral genomes of BTV, BVD and IBR.

### 2.2. BTV-8 Contaminated Semen

Semen was obtained from three bulls that were naturally infected with BTV-8 and had shown clinical signs typical of BT at the end of July in 2007. Semen collection took place three to eight weeks after the last BT clinical signs, at a moment that the bulls were BTV genome and antibody positive. Semen obtained from these bulls was collected, packaged and frozen, as it would be if for commercial use in breeding cattle. BTV-8 was detected and viral load was quantified in batches of semen by both real-time Reverse Transcription-PCR (RT-qPCR) and virus isolation in tissue culture [33]. Four batches of this BTV-8 contaminated semen (including two batches from one of the infected bulls) were kindly provided by Dr E. Dernelle from the AI centre “Association Wallonne de l’Elevage” (AWE, Ciney, Belgium).

### 2.3. Sperm Quality Assessment

Computer-assisted sperm analysis (CASA) was performed using a Hamilton Thorne motility analyzer (CEROS version 12.2c; Hamilton-Thorne Research, Beverly, MA, USA) with the parameter setting as described by Hoflack et al. (2007) [43]. Analysis was based on 20 consecutive images per second to evaluate the percentage motility and the percentage progressive motility. Membrane integrity was evaluated by fluorescence microscopy (400×) following incubation of diluted sperm in fluorescent SYBR-14 (1 µm/mL) and propidium iodide (2.4 µm/mL) for 15 min at 37 °C in the dark (Live-dead kit, Molecular Probes, Eugene, OR, USA). Sperm concentration was determined by means of a Bürker counting chamber and light microscopy (200×). Sperm morphology was assessed by light microscopy (400×) after eosin/nigrosine staining. Spermatozoa were categorized and five subgroups were quantified: (1) normal, (2) abnormal head, (3) abnormal tail, (4) proximal protoplasm droplets, and (5) distal protoplasm droplets.

### 2.4. Study Design

Two independent experiments were performed at two animal facilities with two different teams and at two different times.

#### 2.4.1. Oestrus Synchronization and Artificial Insemination

All heifers were synchronized using two injections with 25 mg dinoprost (Dinolytic^®^, Intervet, Boxmeer, The Netherlands) with an interval of 11 days. All heifers (including controls) displayed signs of oestrus approximately 48 h after the second injection. All heifers, except the control heifers, were inseminated (intra-uterine) with frozen–thawed semen 72 h after the second injection by a veterinarian experienced in performing AI on cattle.

#### 2.4.2. Experiment 1

The first experiment started on 8 March 2010 at the experimental farm of the Faculty of Veterinary Medicine (University of Ghent, Belgium). A total of 12 heifers were segregated into 4 groups (A, B, C, D). Groups A and B were inseminated with two different batches of semen (1.1 and 1.2) from bull 094. Group C received semen batch 2 from bull 202, and group D received semen batch 3 from bull 572. Each heifer received one semen straw of 0.25 mL. Heifer Ctrl-1 was not inseminated and served as a BTV transmission control. Abortion was induced between day 90 and 110 days post-AI (dpai) in pregnant heifers remaining BTV uninfected (determined by BTV RT-qPCR and antibody ELISA) by injecting twice 25 mg dinoprost (prostaglandin F2α, Dinolytic^®^, Intervet, Boxmeer, The Netherlands) per animal with three days interval. Animals were inspected twice a day to collect the foetus for autopsy and testing. Foetal samples were collected for autopsy, histology and BTV RNA detection.

#### 2.4.3. Experiment 2

The second experiment began in September 2010 at the Plate-Forme d’Infectiologie Expérimentale (PFIE) of INRAE in Tours, France, and the animals were housed in a bio-containment level 3 conditioned isolation unit. This experiment consisted of 2 groups of 3 heifers each (E and F) and 1 control heifer. The previous batches 2 and 3 were used to inseminate group E and F heifers. Prior to AI, 15 semen straws from each batch were thawed and pooled to inseminate each heifer with 1 mL of the semen batch and to perform the laboratory tests. Pooling of semen straws is a known practice to compensate for inferior semen quality, such as low progressive sperm motility. Pooling of 4 straws furthermore resulted in a viral load in between the maximum and minimum viral load of the already used semen batches in experiment 1. As in trial one, the control (Ctrl-2) was not inseminated. Abortion was induced in pregnant heifers remaining BTV uninfected, foetuses were collected, autopsy performed, and samples taken as described above.

#### 2.4.4. Pregnancy Diagnosis and Follow-up

The initial diagnosis of pregnancy was performed at 21–22 dpai by measuring progesterone and pregnancy-associated glycoprotein (PAG) in maternal plasma. Progesterone determination on plasma samples was performed using a radio-immuno assay according to Beckers et al. (1975) [44]. A radio-immuno assay (RIA2) using antiserum 706 as described by Perényi et al. (2002) [45] was used to measure PAG in the maternal blood. The detection of progesterone in maternal blood at 21 days gestation (dg) and PAG at 26 dg indicates that an embryo was present in utero at 15 dg. The pregnancy status of each pregnant heifer was continually assessed thereafter by a combination of PAG measurement in maternal blood and ultrasound examination of uterine contents, and the time at which any pregnancy loss occurred was estimated. Sequential heparin blood samples were taken for PAG determination at 24, 26, 28, 31, 35 dpai and afterwards weekly. Samples were centrifuged and plasma was frozen at −80 °C until analysis. Pregnant heifers were monitored on a regular basis by rectal palpation and ultrasound beginning from 28 dpai onwards.

#### 2.4.5. Sampling and Clinical Examination

Clinical examinations, including body temperature monitoring, were performed on the same dates the animals were bled for laboratory diagnosis: before insemination (0) and 3, 7, 10, 14, 17, 21, 24, 28, 31, 35 dpai and afterwards weekly up to 154 dpai. Body temperature above 39.5 °C was defined as febrile. A standardised clinical form for bluetongue signs [46] was completed when the heifers were suspected of BT infection.

#### 2.4.6. Environmental Monitoring and Vector Control

Four CDC black-light traps (model 1212, John W. Hock Company, Gainesville, FL, USA) were installed at the experimental farm of the Faculty of Veterinary Medicine (University of Ghent, Belgium) to monitor the abundance and activity of biting midges (*Culicoides* spp.). Two traps were placed near the animal facilities and two inside the animal housing until the end of June (114 dpai). Weekly captures of two consecutive nights were performed, starting on the 8 March 2010. *Culicoides* species were morphologically identified using the key of Delécolle (1985) [47]. The parous, non-blood engorged female midges were selected as described by Dyce et al. (1969) [48] and pooled for BTV analysis. Heifers that tested positive for BTV by RT-qPCR were immediately separated from the noninfected heifers by moving them to another animal housing with biocontainment level 2 facilities. This animal unit was separated from the outside by a sluice with a CDC black-light trap. From the first week of April, before the vector period was expected to begin, the study animals and all susceptible ruminants in the environment were treated with deltamethrine [49]. The temperature (daily minimum and maximum) was recorded outside in close proximity of both animal housings and inside the animal units.

The second experiment, at the Plate-Forme d’Infectiologie Expérimentale (PFIE) of INRAE Tours France, started in September 2010, when biting midges are very active and, therefore, the animals were housed in a biocontainment level 3 unit.

### 2.5. Bluetongue Virus Laboratory Diagnosis

#### 2.5.1. BTV Genome Detection

Total RNA from 100 µL EDTA-blood was automatically extracted on a JANUS Automated Work-Station (PerkinElmer, Waltham, MA, USA) according to Vandenbussche et al. (2010) [50]. Extraction of RNA from tissue samples (spleen and brain stem) of foetuses was obtained by a mechanical homogenization-lysis step of approximately 40 mg of the tissue sample in 800 µL of TriPure (Invitrogen, Carlsbad, CA, USA) supplemented with synthetic RNA (lambda) of the external control [51] and metallic beads in a 2 mL tube. The samples were shaken 3 times vigorously for 2 min at 20 Hz in a TissueLyser (Qiagen, Hilden, Germany). Chloroform was added (160 µL) and the solution was separated into 3 phases by centrifugation at 12,000 *g* for 15 min at 5 °C. RNA from frozen semen was extracted as described by Vanbinst et al. (2010) [33]. RNA from BTV possibly present in *Culicoides* trapped in the black-light traps (see above) was extracted as described by Vanbinst et al. (2009) [52]. The isolated RNA of all samples was further analysed with the pan-BTV/S5 RT-qPCR according to Vandenbussche et al. (2010) [50], and the result expressed as Cp value. The internal control GAPDH mRNA and the synthetic RNA used as external control were as described by Vandenbussche et al. (2010) [50], except for the *Culicoides*, where 18S rRNA served as an internal control according to Vanbinst et al. (2009) [52].

#### 2.5.2. BTV Antibody Detection

Serum samples were analysed with the ID Screen^®^ Bluetongue Competition assay (ID VET, Montpellier, France) according to the manufacturer’s instructions. Besides the kit controls, a two-fold dilution series of an anti-BTV antibody positive reference serum (CIRAD, Montpellier, France) was included as a working standard in each assay to monitor the performance of the ELISA in real-time. Results were expressed as % negativity (PN) compared to the negative kit control, and transferred to a positive, doubtful or negative result according to cut-off values previously determined by Vandenbussche et al. (2008) [53].

#### 2.5.3. BTV Isolation and Titration

Blood, spleen, brain stem and semen samples were used for virus isolation by intravenous inoculation of embryonated chicken eggs (ECE), as previously described by Vanbinst et al., (2010) [33]. All samples were passaged twice in ECE before being considered negative. Positive isolates from ECE were identified as BTV positive by the pan-BTV/S5 RT-qPCR. The semen samples identified as BTV positive were also isolated on KC cells (Friedrich Loeffler Institute, Greifswald, Germany) [54] and titrated on BHK-21 cells (Friedrich Loeffler Institute, Greifswald, Germany) [33]. Final virus titres were calculated by Kärber’s method [55].

### 2.6. Foetal Examination

A full necropsy was performed of all aborted foetuses that were recovered. Samples of brain, kidney, liver, lung, skeletal muscle, skin, spleen, and thymus were collected from each foetus and prepared for histopathological examination by fixation in formalin for 24 h, processing to paraffin wax, sectioning at 4 µm, and staining with haematoxylin and eosin. For identification of T- and B-lymphocytes, samples were immunolabeled with polyclonal rabbit anti-CD3 antibody (A0452; Dako, Glostrup, Denmark) and polyclonal rabbit anti-CD20 antibody (RB-9013-P; Neomarkers, Fremont, CA, USA), respectively, as described by Vercauteren et al. (2008) [29]. All samples were evaluated histopathologically by light microscopy.

### 2.7. Statistical Analysis

The degree of association between the binary variable (outcome infected or uninfected animal) and the continuous-level variable (Cp value of the semen used) was calculated as described by Delhalle et al. (2008) [56] and expressed as a point–biserial correlation coefficient. The difference between groups of heifers was assessed by a Fisher’s exact test for frequency (number of infected animals by group). The association was further analysed using frequency distributions of infected animals by Cp-classes, as described by Vandenbussche et al. (2008) [53]. Therefore, the heifer groups were pooled according to the dose level of inocula (semen batch) by AI route: (i) Cp-class with values below 21; (ii) Cp-class with values between 21 and 24; and (iii) Cp-class with values higher than 24. Due to the limited number of semen batches, the Cp-classes were chosen arbitrarily. The difference between the pools (groups of heifers) was assessed with a nonparametric Wilcoxon rank sum test for quantitative parameters (dpai of first positive RT-qPCR, dpai of first positive ELISA and dpai of abortion). For all tests, *p* values < 0.05 were considered as significant [57].

## 3. Results

### 3.1. Animals

All animals were negative for BTV, BVD, IBR, EBL, Neospora and Q Fever antibodies, and negative for the presence of viral genome of BTV, BVD and IBR before transport from Ireland and before AI (0 dpai) in both experimental centres.

### 3.2. Real-Time RT-PCR Analysis and Viral Titration of the Semen Batches

The viral load in each batch of semen after it was thawed was determined by both RT-qPCR (Cp value) and virus isolation in tissue culture (titre of infectious virus). The Cp value (Table 1) determined in the semen inseminated in the first experiment (0.25 mL) was between 20.5 and 27.2 per semen straw of 0.25 mL. The presence of live virulent BTV was confirmed in all semen batches by isolation on ECE and on KC cells. The BTV titre on BHK-21 cells was between 10^4.9^ and 10^2.7^ TCID50. The viral load of batches 2 and 3 used in the second experiment was determined by RT-qPCR per 1 mL of inseminated semen and had Cp values of 23.4 and 25.2, respectively. The viral titre of the pooled aliquot of 1 mL was 10^3.3^ (batch 2) and 10^3.4^ TCID50 (batch 3), and, as such, approximately four times the doses of a single semen straw.

### 3.3. Sperm Quality Assessment

Sperm motility determined by CASA varied between 7% and 29% for progressive motility and 26% and 56% for total motility (Table 2). The total sperm concentration after thawing was between 92 million and 124 million sperm cells/mL. The percentage of viable sperm after SYBR-14/PI staining was between 25% and 44%. Between 63% and 82% of the viable sperm cells were categorized as having a normal morphology. Although all these characteristics indicate that the used ejaculates had diminished sperm quality, the conception rate was high.

### 3.4. Experiment 1

**Figure 1:**
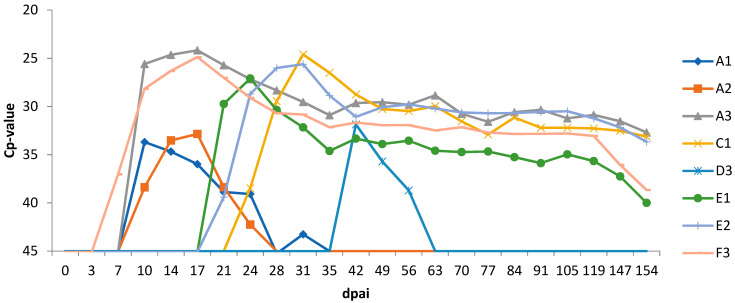
RNAemia as determined by real-time RT-PCR detection of BTV genome in blood of heifers infected with BTV-8 contaminated semen from natural-infected bulls. dpai: days post artificial insemination.

**Figure 2:**
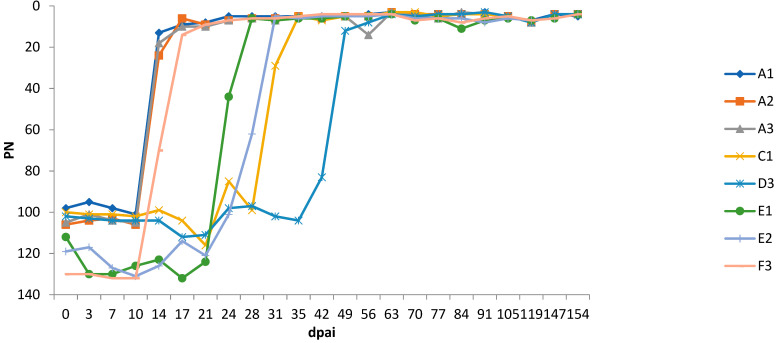
The level of antibodies in blood of heifers infected with BTV-8 contaminated semen from natural-infected bulls, as determined by a competitive ELISA and expressed in percentage negativity (PN). dpai: days post artificial insemination.

**Figure 3:**
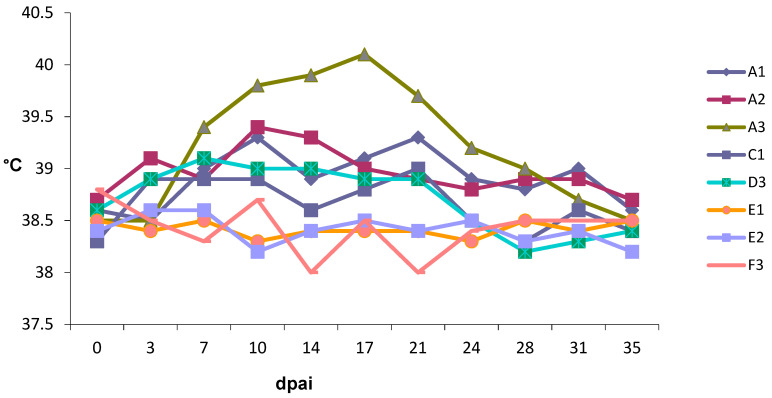
The body temperature of heifers infected with BTV-8 contaminated semen from natural-infected bulls until 35 days post-artificial insemination (dpai); thereafter, temperature remained normal.

All 12 heifers had blood levels of progesterone and PAG at 22 dpai, consistent with pregnancy. In group A (Table 1) all heifers were considered BTV infected as they became positive for BTV genome at 10 dpai by RT-qPCR (Figure 1) and for BTV antibodies at 14 dpai by ELISA. All remained seropositive at a high level until the end of the trial (Figure 2). Heifers A1 and A2 showed short and mild viraemia, as determined by RT-qPCR, reaching a viral RNA peak after a couple of days and clearing the virus from the blood in two weeks’ time (Figure 1). Heifer A3, on the other hand, showed a strong and long-lasting viraemia, and BTV could be isolated on ECE at 10 dpai. The RT-qPCR peaked between 10 and 17 dpai, then the amount of viral RNA decreased slowly and remained stable at around 30 Cp. Clinical signs of BT were very mild, including a slight increase in body temperature in several animals, but heifer A3 was the only febrile animal with a body temperature rising above 39.5 °C for several days (Figure 3). Other clinical signs were serous nasal discharge (several animals), oedema of the legs (heifer A1) and mild submandibular oedema (heifer A3). Pregnancy loss occurred early in each of the heifers from A1–A3 (Table 1). Most likely, early embryonic death (EED) was followed by resorption.

In group B, which received a 30-times lower viral dose than group A, pregnancy was maintained throughout the study period in all heifers, with no evidence of infection, remaining clinically normal and negative for viral genome and antibody throughout the study. Neither the gross pathology nor the histopathology could identify any abnormality in the organs or tissues of the foetuses recovered after induced expulsion. BT viral genome was not detected in foetal spleen or brainstem.

In group C, which received a similar viral dose as group B, BTV RNA was found in the blood of one heifer (heifer C1; Table 1) at 24 dpai and antibodies were detected in serum from 31 dpai (Figure 1 and Figure 2). This heifer showed a similar strong and long-lasting viraemia to that seen in heifer A3 but starting 2 weeks later. BTV could be isolated at 28 dpai and the animal also remained seropositive at a high level. Early pregnancy loss (40 dpai) occurred and the foetus was likely resorbed. Unlike heifer A3, this heifer had no clinical signs of BT disease. The other 2 heifers were healthy, no lesions were observed in tissues of their experimentally aborted foetuses, and viral genome was not detected in foetal spleen or brainstem.

In group D, which received the lowest viral dose, only heifer D3 developed a BTV-8 infection (Table 1). This happened at a later stage, with viral genome detected from 42 dpai and antibodies from 49 dpai. This heifer experienced a short, mild viraemia (Figure 1), similar to those seen in heifers A1 and A2, and was seropositive until the end of the trial (Figure 2). Heifer D3 showed no clinical signs and gave birth to a normal full-term calf that tested negative for BTV viral genome and antibody. The other two heifers (D1 and D2) were also clinically unaffected and tested negative for viral genome and antibody, but pregnancy loss occurred in heifer D2 at 56 dpai. Foetuses were obtained for examination from heifer D1 (after induced expulsion) and heifer D2 (after spontaneous expulsion) but no lesions were observed and the virus was not detected in foetal organs. The control animal (heifer Ctrl-1) remained negative in BTV RT-qPCR and antibody tests throughout the whole experiment.

### 3.5. Experiment 2

Heifers E1, E2, E3, F1 and F2 conceived after insemination with 1 mL of BTV-8 contaminated semen. Heifer F3 had a negative progesterone and PAG result at 22 dpai, indicating no conception or early embryonic death. In group E (Table 1), heifers E1 and E2, were positive in RT-qPCR from 21 dpai (Figure 1) and BTV was isolated at 24 and 28 dpai, respectively. The serological test was positive from 24 dpai and 29 dpai, respectively (Figure 2). Heifer F3 from group F became viraemic at 7 dpai, 14 days earlier than heifers E1 and E2, and BTV was isolated by ECE inoculation at 14 dpai (Table 1). Antibodies were detected from 17 dpai. The three BT infected heifers (E1, E2, F3) developed strong viraemia and high antibody levels lasting until the end of the study (Figure 1 and Figure 2), but were clinically unaffected, except for lesions on the muzzle of heifer E2. Heifer E1 aborted at 49 dpai and the foetus was likely resorbed. Heifer E2 delivered a healthy calf at term that was negative for BTV by RT-qPCR and ELISA. Heifer E3, F1 and F2 were healthy, no lesions were observed in tissues of their experimentally aborted foetuses, and viral genome was not detected in foetal spleen or brainstem. The control animal (heifer Ctrl-2) remained negative in BTV RT-qPCR and antibody tests.

### 3.6. Analysis of the Culicoides Trapped

A total of 310 *Culicoides* were captured near the animal facility in experiment 1, of which 245 were parous, nonblood engorged females and, thus, potential vectors. The latter were collected in all months (March (*n* = 3), April (*n* = 27), May (*n* = 53), June (*n* = 162)), with a weekly average of 14 potential vectors (*n* = 17, range = 0–47). Parous midges of the *obsoletus* complex, including 185 *C. obsoletus/scoticus*, 12 *C. dewulfi* and 41 *C. chiopterus*, and seven parous *C. punctatus* were found. A total of 14 pools of parous, nonblood engorged female midges (with a maximum of 10 midges per pool) were analysed using the pan-BTV/S5 RT-qPCR, with negative results. The second RT-qPCR targeting *Culicoides* 18S rRNA as an internal RNA quality control allowed us to validate the outcome of all BTV tested pools. In the animal facility, a total of 11 *Culicoides* (all of the *obsoletus* complex), with 4 and 2 parous, nonblood engorged female *C. obsoletus/scoticus* and *C. chiopterus*, respectively, were collected in May (*n* = 1) and June (*n* = 5). These captures tested negative for the BTV genome but the amount of RNA was probably too low to get a positive result for the internal control. The captures from the CDC black-light trap in the sluice were not analysed.

### 3.7. Statistical Analysis

The point–biserial correlation coefficient between the outcome of the animal (infected versus uninfected) and the Cp value was −0.62 (*p*-value = 0.014), which indicates a significant association between the outcome and the reverse of the Cp value. Indeed, the number of infected animals per group decreased significantly as function of the viral load, expressed as Cp level, of the inocula administered by AI route (Fisher’s exact test; *p* = 0.043). The number of days which elapsed between AI and the onset of viraemia, determined by RT-qPCR, was significantly less in the pool of infected animals with inocula with Cp value below 21 than in the pool of infected animals with inocula with a Cp value between 21 and 24 (Wilcoxon rank sum test; *p* = 0.046). The same trend was observed for the number of days which elapsed between AI and the onset of seroconversion, determined by ELISA, being significantly less in the group infected animals with inocula of Cp value below 21 than in the pool of infected animals with an inocula having a Cp value above 24 (Wilcoxon rank sum test; *p* = 0.037). Comparing the pools with a Cp value below 21 with Cp 21–24 for the number of dpai of first positive ELISA yielded a *p* = 0.053 that was just above the cut off value for significance. For the stage of gestation at which pregnancy loss was estimated to have occurred (abortion), only one observation was performed in the pool with a Cp value between 21 and 24. After this observation was combined with the pool with a Cp value above 24, the abortion delay was significantly more important for this latter pool (Wilcoxon rank sum test; *p* = 0.046). All other statistical comparisons were not significantly different.

## 4. Discussion

Bulls that are persistently infected with BTV, following intrauterine infection, have been found to shed the virus intermittently in their semen and are presumed to do so on a lifelong basis [58,59,60]. One such bull transmitted infection to cows with which it mated, resulting in the birth of twelve calves that were viraemic and had suffered severe teratogenesic effects [58]. However, MacLachlan et al. (1990) [61] raised doubts about the persistence of BTV infection in ruminants, in light of the normal pathogenesis of bluetongue. Pascall et al. (2020) [20] expressed serious questions about the possibility of long-term persistence of BTV in the susceptible population as a possible mechanism to account for the re-emergence of BTV-8 in France in 2015 [18], several years after the 2006–2010 epizootic in West-Central Europe. BTV has been experimentally transmitted by AI using tissue culture adapted strains of BTV serotypes 10, 11, 13 and 17 [37,38]. However, BTV shedding in the semen of naturally infected bulls was considered to be extremely rare [62], if at all possible [40,63,64], and there are no previous reports of a field strain of BTV being transmitted between cattle via semen. Therefore, the suggestion of Pascall et al. (2020) [20] that frozen bovine semen might have been the source of re-emergent BTV-8 required experimental demonstration that BTV infection could be transmitted between cattle by AI with BTV-contaminated semen. As BTV-8 was detected in semen of bulls during the 2006–2010 epidemic [33], the aim of the current study was to address this knowledge gap on the possibility of BTV transmission through semen.

The viral titres in the frozen semen used in previous studies mentioned above were comparable to the viral titres in the present study, as were the BTV transmission rates: 3 of 9 [37] and 2 of 4 [38] compared to 8 of 18 heifers becoming infected in the present study. The virus was not isolated from any of the foetuses in those previous studies, as was the case with the foetuses that were expelled before term and recovered in the present study. Pregnancy loss in those infected heifers from which a foetus was not recovered may be attributable to BTV-8 infection, as reproductive failure in cattle was a feature of the BTV-8 epidemic 2006–2010 [31]. In the present study, both infected heifers that brought their pregnancy to full-term (D3, E2) gave birth to normal, healthy calves that tested negative for both BTV genome and antibody. While this contrasts with the pattern of congenital infection and malformation in calves born to infected dams during the 2006–2010 BTV-8 epidemic [29,31] and the re-emerging disease in France in the period 2018–2019 [32], it could simply be an artefact of the small numbers (only two full-term calves) available for examination in the present study, or could be expected given the large variation also seen in the field after a bluetongue infection.

Intradermal, subcutaneous, and intravenous routes are the most frequently used inoculation methods for experimental infection. When a BTV-11 strain grown on insect cells was inoculated intradermally and subcutaneously into four cows, all four became viraemic, whereas none of four cows receiving an intra-uterine inoculation of the same virus developed viraemia [60]. However, in the present study, viraemia was evident in a total of 8 out of 18 heifers that were challenged by AI in two independent experiments; 5 of 12 animals became infected in the first experiment and 3 of 6 in the second. In addition to the variation observed within each group, statistically significant differences were observed between groups exposed to different doses of virus. The most notable difference between groups was the proportion of animals that became infected. The transmission rate in group A, which received semen with the highest viral load, was significantly higher than in groups B, C, D and F, following AI with semen which had a lower viral load. Group E, where semen was used with an intermediate Cp value, had a transmission rate in between group A and the other infected groups. The semen batches with a Cp value belonging to the pool above 24 gave rise to one BTV infected animal per group, except in group B. This viral load could be at the “cut off” level for transmission and could explain the absence of infection in Group B.

Variations in pathogenesis, duration and clinical outcome of BTV-infections have been observed among individual animals, but the underlying mechanisms are still not fully understood [65,66,67]. Despite the fact that the animals in this study were of similar age and breed, and within the groups all subject to the same treatment, they did respond differently to the virus. Some of the infected animals had BT clinical signs but most had none. The infected heifers either had a long-term viraemia with a plateau phase after a peak viraemia, or a short-term viraemia without a plateau phase. No statistical relationship could be revealed between the clinical signs or the length of the viraemia and the viral load in the semen used for AI. The lack of clinical signs in the dam does not preclude the development of severe lesions in internal organs of calves that are infected in utero, as has been shown in experimental infections with a low passage isolate of the European BTV-8 strain causing damage to the endothelial tissues [46,68]. All eight heifers that became infected in the present study developed and maintained high antibody levels, regardless of whether or not they had shown clinical signs or prolonged viraemia.

In contrast to subcutaneous, intradermal or intravenous inoculation [4,60,68], substantial variation in onset of viraemia and onset of antibody formation were observed in this study after intra-uterine infection, being significantly related to the level of viral contamination of the semen batches used. Wild-type BTV targets endothelial cells and mononuclear phagocytes [2] but not normally endometrial epithelial cells. However, AI in this study was done at oestrus, transforming the uterus wall to a thickened endometrium with more blood vessels [69]. The difference in viral load and the variable number of accessible blood vessels and endothelial cells could explain the variation in the onset of infections or even the inability to infect some of the heifers (Group B). The difference in the attack rate of the intra-uterine blood vessels, and, consequently, the variation in the endometrium condition for the development of the embryo, could also explain the significant difference in the ratio and the time of abortion. Another factor that could play a role in the variation in the onset of infection is a possible prolonged stay of the virus in the uterus, as described for CSF [70].

The fact that the virus could be isolated from the blood of several heifers shows that these animals were contagious to the *Culicoides* spp., which could further transmit the virus, supporting the hypothesis advanced by Pascall et al. (2020) [19]. Probably, BTV could not be isolated from heifers A1, A2 and D3 because the viral load at peak viraemia was too low, as described previously [23]. Competent Authorities (CA) and the agriculture industry should work together to increase awareness campaigns, especially for AI Centres, to prevent the nonintentional misuse of preserved semen and to assure that all international standards [8,71] and regulations [72,73] are implemented.

The question of whether BTV transmission in the experimental heifers of the present study could have occurred by any means other than exposure to contaminated semen by AI should not be avoided. The experimental animals obtained from Ireland were known not to have been infected with BT in 2010 or before. The inseminations in the first experiment were performed at the beginning of March 2010, during the vector-free period (when vector activity is very low) that started in Belgium on 14 December 2009 and ended on 3 May 2010 [74]. Although vector transmission during this time of the year is unlikely to occur due to adverse climate conditions for BTV replication in the vector, the study animals were treated with deltamethrine [49]; four black-light traps were installed near and inside the animal facilities and the *Culicoides* checked were BTV RT-qPCR negative. All animals that developed a BTV viraemia were immediately moved to another animal housing with a sluice and black-light trap. The last infection in this experiment, detected by RT-qPCR, was on 19 April (42 dpai), well before the end of the vector-free period. Before 42 dpai the average temperature measured inside and outside the animal housing was always below 12 °C. Given these conditions, virus replication within a vector and subsequent transmission to a new vertebrate host is excluded [75]. In addition, a bluetongue sentinel surveillance program and cross-sectional serological survey in cattle in the period 2010–2011 proved that Belgium had been free from BTV since 2010 [15]. Although many precautions were taken, a low, albeit still present, risk that naïve *Culicoides* could have blood-fed on the infected cattle remains and, therefore, prior to commencing experiment 1, a risk assessment was undertaken as to the level of biocontainment required. Taking into account the mitigation measures mentioned above, the fact that there was compulsory BTV-8 vaccination using an inactivated BTV-8 vaccine of the entire cattle and sheep population of Belgium at that time, and that this vaccination program had been in place since Spring 2008, the biocontainment of the animal facility was considered to be sufficient to prevent any escape of bluetongue virus and/or further transmission of bluetongue. The heifers from the second experiment were isolated throughout the experiment in insect-proof animal housing with biocontainment level 3. All materials used for insemination, blood collection and pregnancy control were strictly separated between individual animals preventing any form of iatrogenic or horizontal transmission. The conclusion that all BTV infections in this study were caused by BTV contaminated semen and that no secondary infections occurred in both trials is also supported by the fact that the control animals in both experiments remained negative in RT-qPCR and serology throughout the study.

## 5. Conclusions

These two separate experiments showed that a BTV-8 field strain could be transmitted relatively efficiently to heifers previously unexposed to BTV by AI using BTV-contaminated frozen–thawed semen, collected as per commercial cattle breeding practice, from bulls that were naturally infected with BTV-8. This supports the suggestion of Pascall et al. (2020) [20] that the re-emergence of BTV-8 in France in 2015 may be attributable to the use of BTV-contaminated bovine semen that had been collected during the 2006–2010 epizootic. In the present study, a statistically significant association was found between the viral load of the semen used for AI and the efficiency of transmission of infection, the time of onset of viremia post AI, and the stage at which pregnancy loss was estimated to have occurred. These findings are consistent with some of the features of BTV-8 infection seen in Europe that are considered unusual for bluetongue. It should not be presumed that the same pattern would pertain to other field strains of BTV without performing an experimental evaluation such as described in this paper.

## Figures and Tables

**Table 1:** Artificial insemination of heifers with bluetongue virus serotype 8 (BTV-8) contaminated semen. Overview of (i) two independent experiments; (ii) heifer groups; (iii) the semen batches used; (iv) heifer ID; (v) days post artificial insemination (dpai), at which the heifers became BTV real-time RT-PCR positive; (vi) dpai at which BTV was isolated; (vii) dpai at which the heifers became serological positive (competitive antibody-ELISA); and (viii) time points in dpai of pregnancy loss estimated using PAG and ultrasound results.

	Heifer Group	Semen Batch (Bull ID)	Semen RT-qPCR Cp-Value	Heifer ID	Dpai First BTV Genome Detection	Dpai First BTV Isolation Positive	Dpai First BTV Antibody Detection	Pregnancy Loss (Dpai)
Exp 1-AI with 0.25 mL semen	A	1.1 (094)	20.5	A1	10	neg	14	35
				A2	10	neg	14	26
				A3	10	10	14	26
	B	1.2 (094)	25.1	B1	neg	nd	neg	No
				B2	neg	nd	neg	No
				B3	neg	nd	neg	No
	C	2 (202)	25.4	C1	24	28	31	40
				C2	neg	nd	neg	No
				C3	neg	nd	neg	No
	D	3 (572)	27.2	D1	neg	nd	neg	No
				D2	neg	nd	neg	56
				D3	42	neg	49	No
	Ctrl	na		Ctrl-1	neg	nd	neg	na
Exp 2-AI with 1 mL semen	E	2 (202)	23.4	E1	21	24	24	49
				E2	21	28	29	No
				E3	neg	nd	neg	No
	F	3 (572)	25.2	F1	neg	nd	neg	No
				F2	neg	nd	neg	No
				F3	7	14	17	*
	Ctrl	na		Ctrl-2	neg	nd	neg	na

Exp: Experiment; Ctrl: control animal; na: not applicable; nd: not done; dpai: days post artificial insemination; neg: animal remained negative for the test applied; No: no pregnancy loss during the trial; * Pregnancy diagnosis was negative at 22 dpai.

**Table 2:** Quality assessment of four BTV-contaminated semen batches from three naturally BTV-infected bulls.

		Sperm Quality				
Bull ID	Semen Batch	Progressive Motility (%)	Total Motility (%)	Concentration (× 10^6^ Sperm cells/mL)	Viable (%)	Normal Morphology (%)
094	1.1	27	47	124	44	82
094	1.2	29	56	104	44	80
202	2	16	33	94	25	70
572	3	7	26	92	42	63

ID: identification number.

## Data Availability

The main data presented in this study are available within the study itself and other data may be made available through contact with the corresponding author.

## References

[1] Mertens P.P., Diprose J., Maan S., Singh K.P., Attoui H., Samuel A.R. (2004). Bluetongue virus replication, molecular and structural biology. Vet. Ital..

[2] Barrat-Boyes S.M., MacLachlan N.J. (1994). Dynamics of viral spread in bluetongue virus infected calves. Vet. Microbiol..

[3] Orange J.P., Dinh E.T.N., Goodfriend O., Citino S.B., Wisely S.M., Blackburn J.K. (2021). Evidence of epizootic hemorrhagic disease virus and bluetongue virus exposure in nonnative ruminant species in northern Florida. J. Zoo Wildl. Med..

[4] Bonneau K.R., DeMaula C., Mullens B., MacLachlan N.J. (2002). Duration of viraemia infectious to *Culicoides sonorensis* in bluetongue virus-infected cattle and sheep. Vet. Microbiol..

[5] Linden A., Gregoire F., Nahayo A., Hanrez D., Mousset B., Massart A.L., De Leeuw I., Vandemeulebroucke E., Vandenbussche F., De Clercq K. (2010). Bluetongue virus in wild deer, Belgium, 2005–2008. Emerg. Infect. Dis..

[6] López-Olvera J.R., Falconi C., Férnandez-Pacheco P., Fernández-Pinero J., Sánchez M.A., Palma A., Herruzo I., Vicente J., Jiménez-Clavero M.A., Arias M. (2010). Experimental infection of European red deer (*Cervus elaphus*) with bluetongue virus serotype 1 and 8. Vet. Microbiol..

[7] Gethmann J., Probst C., Conraths F.J. (2020). Economic Impact of a Bluetongue Serotype 8 Epidemic in Germany. Front. Vet. Sci..

[8] World Organization for Animal Health (OIE) (2020). Terrestrial Animal Health Code, Chapter 8.3: Infection with Bluetongue Virus. https://www.oie.int/index.php?id=169&L=0&htmfile=chapitre_bluetongue.htm.

[9] Toussaint J.F., Sailleau C., Mast J., Houdart P., Czaplicki G., Demeestere L., Vandenbussche F., Van Dessel W., Goris N., Breard E. (2007). Bluetongue in Belgium, 2006. Emerg. Infect. Dis..

[10] Saegerman C., Berkvens D., Mellor P.S. (2008). Bluetongue epidemiology in the European Union. Emerg. Infect. Dis..

[11] Zientara S., MacLachlan N.J., Calistri P., Sanchez-Vizcaino J.M., Savini G. (2010). Bluetongue vaccination in Europe. Expert Rev. Vaccines.

[12] Wilson A., Carpenter S., Gloster J., Mellor P. (2007). Re-emergence of bluetongue in northern Europe in 2007. Vet. Rec..

[13] Backx A., Heutink R., van Rooij E., van Rijn P. (2009). Transplacental and oral transmission of Wild-type bluetongue virus serotype 8 in cattle after experimental infection. Vet. Microbiol..

[14] Méroc E., Herr C., Verheyden B., Hooyberghs J., Houdart P., Raemaekers M., Vandenbussche F., De Clercq K., Mintiens K. (2009). Bluetongue in Belgium: Episode II. Transbound. Emerg. Dis..

[15] Vangeel I., De Leeuw I., Méroc E., Vandenbussche F., Riocreux F., Hooyberghs J., Raemaekers M., Houdart P., Van der Stede Y., De Clercq K. (2012). Bluetongue sentinel surveillance program and cross-sectional serological survey in cattle in Belgium in 2010–2011. Prev. Vet. Med..

[16] Sailleau C., Bréard E., Viarouge C., Vitour D., Romey A., Garnier A., Fablet A., Lowenski S., Gorna K., Caignard G. (2017). Re-Emergence of Bluetongue Virus Serotype 8 in France, 2015. Transbound. Emerg. Dis..

[17] European Commission, DG Health and Consumers, Animal Health and Welfare. Bluetongue, Control Measures. https://ec.europa.eu/food/sites/food/files/animals/docs/ad_control-measures_bt_restrictedzones.pdf.

[18] Courtejoie N., Durand B., Bournez L., Gorlier A., Bréard E., Sailleau C., Vitour D., Zientara S., Baurier F., Gourmelen C. (2018). Circulation of bluetongue virus 8 in French cattle, before and after the re-emergence in 2015. Transbound. Emerg. Dis..

[19] Bréard E., Sailleau C., Quenault H., Lucas P., Viarouge C., Touzain F., Fablet A., Vitour D., Attoui H., Zientara S. (2016). Complete Genome Sequence of Bluetongue Virus Serotype 8, Which Reemerged in France in August 2015. Genome Announc..

[20] Pascall D.J., Nomikou K., Bréard E., Zientara S., Filipe A.D.S., Hoffmann B., Jacquot M., Singer J.B., De Clercq K., Bøtner A. (2020). “Frozen evolution” of an RNA virus suggests accidental release as a potential cause of arbovirus re-emergence. PLoS Biol..

[21] Thiry E., Saegerman C., Guyot H., Kirten P., Losson B., Rollin F., Bodmer M., Czaplicki G., Toussaint J.F., De Clercq K. (2006). Bluetongue in northern Europe. Vet. Rec..

[22] Elbers A.R., Backx A., Mintiens K., Gerbier G., Staubach C., Hendrickx G., van der Spek A. (2008). Field observations during the Bluetongue serotype 8 epidemic in 2006. II. Morbidity and mortality rate, case fatality and clinical recovery in sheep and cattle in the Netherlands. Prev. Vet. Med..

[23] Zanella G., Martinelle L., Guyot H., Mauroy A., De Clercq K., Saegerman C. (2013). Clinical pattern characterization of cattle naturally infected by BTV-8. Transbound. Emerg. Dis..

[24] De Clercq K., De Leeuw I., Verheyden B., Vandemeulebroucke E., Vanbinst T., Herr C., Meroc E., Bertels G., Steurbaut N., Miry C. (2008). Transplacental infection and apparently immunotolerance induced by a wild-type bluetongue virus serotype 8 natural infection. Transbound. Emerg. Dis..

[25] Menzies F.D., McCullough S.J., McKeown I.M., Forster J.L., Jess S., Batten C., Murchie A.K., Gloster J., Fallows J.G., Pelgrim W. (2008). Evidence for transplacental and contact transmission of bluetongue virus in cattle. Vet. Rec..

[26] Saegerman C., Bolkaerts B., Baricalla C., Raes M., Wiggers L., de Leeuw I., Vandenbussche F., Zimmer J.Y., Haubruge E., Cassart D. (2011). The impact of naturally-occurring, trans-placental bluetongue virus serotype-8 infection on reproductive performance in sheep. Vet. J..

[27] Richardson C., Taylor W.P., Terlecki S., Gibbs E.P.J. (1985). Observations on transplacental infection with bluetongue virus in sheep. Am. J. Vet. Res..

[28] Richards W.P., Crenshaw G.L., Bushnell R.B. (1971). Hydranencephaly of calves associated with natural bluetongue virus infection. Cornell Vet..

[29] Vercauteren G., Miry C., Vandenbussche F., Ducatelle R., Van der Heyden S., Vandemeulebroucke E., De Leeuw I., Deprez P., Chiers K., De Clercq K. (2008). Bluetongue virus serotype 8-associated congenital hydranencephaly in calves. Transbound. Emerg. Dis..

[30] Desmecht D., Bergh R.V., Sartelet A., Leclerc M., Mignot C., Misse F., Sudraud C., Berthemin S., Jolly S., Mousset B. (2008). Evidence for transplacental transmission of the current wild-type strain of bluetongue virus serotype 8 in cattle. Vet. Rec..

[31] Darpel K.E., Batten C.A., Veronesi E., Williamson S., Anderson P., Dennison M., Clifford S., Smith C., Philips L., Bidewell C. (2009). Transplacental transmission of bluetongue virus 8 in cattle, UK. Emerg. Infect. Dis..

[32] Vinomack C., Rivière J., Bréard E., Viarouge C., Postic L., Zientara S., Vitour D., Belbis G., Spony V., Pagneux C. (2020). Clinical cases of Bluetongue serotype 8 in calves in France in the 2018–2019 winter. Transbound. Emerg. Dis..

[33] Vanbinst T., Vandenbussche F., Dernelle E., De Clercq K. (2010). A duplex real-time RT-PCR for the detection of bluetongue virus in bovine semen. J. Virol. Methods.

[34] Leemans J., Raes M., Vanbinst T., De Clercq K., Saegerman C., Kirschvink N. (2012). Viral RNA load in semen from bluetongue serotype 8-infected rams: Relationship with sperm quality. Vet. J..

[35] Kirschvink N., Raes M., Saegerman C. (2009). Impact of a natural bluetongue serotype 8 infection on semen quality of Belgian rams in 2007. Vet. J..

[36] Müller U., Kemmerling K., Straet D., Janowitz U., Sauerwein H. (2009). Effects of bluetongue virus infection on sperm quality in bulls: A preliminary report. Vet. J..

[37] Bowen R., Howard T., Pickett B. (1985). Seminal shedding of bluetongue virus in experimentally infected bulls. Prog. Clin. Biol. Res..

[38] Thomas F.C., Singh E., Hare W. (1985). Embryo transfer as a means of controlling viral infections. VI. Bluetongue-virus free calves from infectious semen. Theriogenology.

[39] Kirkland P.D., Hawkes R.A. (2004). A comparison of laboratory and ‘wild’ strains of bluetongue virus-is there any difference and does it matter?. Vet. Ital..

[40] Kirkland P.D., Melville L., Hunt N., Williams C., Davis R. (2004). Excretion of bluetongue virus in cattle semen: A feature of laboratory-adapted virus. Vet. Ital..

[41] Savini G., Lorusso A., Paladini C., Migliaccio P., Di Gennaro A., Di Provvido A., Scacchia A.M., Monaco F. (2014). Bluetongue serotype 2 and 9 modified live vaccine viruses as causative agents of abortion in livestock: A retrospective analysis in Italy. Transbound. Emerg. Dis..

[42] Napp S., Allepuz A., Garcia-Bocanegra I., Alba A., Vilar M.J., Casal J. (2011). Quantitative assessment of the probability of bluetongue virus transmission by bovine semen and effectiveness of preventive measures. Theriogenology.

[43] Hoflack G., Opsomer G., Rijsselaere T., Van Soom A., Maes D., de Kruif A., Duchateau L. (2007). Comparison of computer-assisted sperm motility analysis parameters in semen from belgian blue and Holstein-Friesian bulls. Reprod. Dom. Anim..

[44] Beckers J.-F., Ballman P., Ectors F., Derivaux J., Herlant M.M. (1975). Le dosage radio-immunologique de la progesterone plasmatique chez la vache. C. R. Acad. Hebd. Seances Acad. Sci. D.

[45] Perényi Z.S., Szenci O., Sulon J., Drion P.V., Beckers J.-F. (2002). Comparison of the ability of three radioimmunoassay to detect pregnancy-associated glycoproteins in bovine plasma. Reprod. Dom. Anim..

[46] Dal Pozzo F., De Clercq K., Guyot H., Vandemeulebroucke E., Sarradin P., Vandenbussche F., Thiry E., Saegerman C. (2009). Experimental reproduction of bluetongue virus serotype 8 clinical disease in calves. Vet. Microbiol..

[47] Delécolle J.C. (1985). Nouvelle Contribution à l’Etude Systématique et Iconographique des Espèces du Genre Culicoides (Diptera: Ceratopogonidae) du Nord-Est de la France. Ph.D. Thesis.

[48] Dyce A.L. (1969). The recognition of nulliparous and parous Culicoides (Diptera: Ceratopogonidae) without dissection. J. Aust. Entomol. Soc..

[49] Mullens B.A., Gerry A., Monteys V., Pinna M., González A. (2010). Field studies on Culicoides (Diptera: Ceratopogonidae) activity and response to deltamethrin applications to sheep in northeastern Spain. J. Med. Entomol..

[50] Vandenbussche F., Vandemeulebroucke E., De Clercq K. (2010). Simultaneous detection of bluetongue virus RNA, internal control GAPDH mRNA, and external control synthetic RNA by multiplex real-time PCR. Methods Mol. Biol..

[51] Vandemeulebroucke E., De Clercq K., Van der Stede Y., Vandenbussche F. (2010). Proposal of a validation method for automated nucleic acid extraction and RT-qPCR analysis: An example with Bluetongue virus. J. Virol. Methods.

[52] Vanbinst T., Vandenbussche F., Vandemeulebroucke E., De Leeuw I., Deblauwe I., De Deken G., Madder M., Haubruge E., Losson B., De Clercq K. (2009). Bluetongue Virus Detection by Real-Time RT-PCR in Culicoides Captured During the 2006 Epizootic in Belgium and Development of an Internal Control. Transbound. Emerg. Dis..

[53] Vandenbussche F., Vanbinst T., Verheyden B., Van Dessel W., Demeestere L., Houdart P., Bertels G., Praet N., Berkvens D., Mintiens K. (2008). Evaluation of antibody-ELISA and real-time RT-PCR for the diagnosis and profiling of bluetongue virus serotype 8 during the epidemic in Belgium in 2006. Vet. Microbiol..

[54] Mertens P.P., Burroughs J.N., Walton A., Wellby M.P., Fu H., O’Hara R.S., Brookes S.M., Mellor P.S. (1996). Enhanced infectivity of modified bluetongue virus particles for two insect cell lines and for two Culicoides vector species. Virology.

[55] Kärber G., Lennette E.H., Schmidt N.J. (1979). Calculation of the LD50 titer by Kärber method. Diagnostic Procedures for Viral Rickettsial and Chlamydial Infections.

[56] Delhalle L., De Sadeleer L., Bollaerts K., Farnir F., Saegerman C., Korsak N., Dewulf J., De Zutter L., Daube G. (2008). Risk factors for Salmonella and hygiene indicators in the 10 largest Belgian pig slaughterhouses. J. Food Prot..

[57] Petrie A., Watson P. (2013). Statistics for Veterinary and Animal Science.

[58] Luedke A.J., Walton T.E. (1981). Effect of natural breeding of heifers to a bluetongue virus carrier bull. Bov. Pract..

[59] Schultz R.D., Rhodes P., Panangala V.S., Kaproth D. (1985). Unusual observations on a serologically seronegative bluetongue virus infected bull. Prog. Clin. Biol. Res..

[60] Parsonson I.M., Thompson L.H., Walton T.E. (1994). Experimentally induced infection with bluetongue virus serotype 11 in cows. Am. J. Vet. Res..

[61] MacLachlan N.J., Jagels G., Rossitto P.V., Moore P.F., Heidner H.W. (1990). The pathogenesis of experimental bluetongue virus infection of calves. Vet. Pathol..

[62] Osburn B.I. (1994). The impact of bluetongue virus on reproduction. Comp. Immunol. Microbiol. Infect. Dis..

[63] Phillips R.M., Carnahan D.L., Rademacher D.J. (1986). Virus isolation from semen of bulls serologically positive for bluetongue virus. Am. J. Vet. Res..

[64] Gard G.P., Melville L.F., Shorthose J.E. (1989). Investigations of bluetongue and other arboviruses in the blood and semen of naturally infected bulls. Vet. Microbiol..

[65] Brodie S.J., Wilson W.C., O’Hearn P.M., Muthui D., Diem K., Pearson L.D. (1998). The effects of pharmacological and Lentivirus-induced immune suppression on Orbivirus pathogenesis: Assessment of virus burden in blood monocytes and tissues by reverse-transcription-In situ PCR. J. Virol..

[66] Hemati B., Contreras V., Urien C., Bonneau M., Takamatsu H.H., Mertens P.P., Bréard E., Sailleau C., Zientara S., Schwartz-Cornil L. (2009). Bluetongue targets conventional dendritic cells in skin lymph. J. Virol..

[67] Maclachlan N.J., Mayo C.E., Daniels P.W., Savini G., Zientara S., Gibbs E.P. (2015). Bluetongue. Rev. Sci. Tech..

[68] Darpel K., Batten C., Veronesi E., Shaw A., Anthony S., Bachenek-Bankowska K., Kgosana L., Bin-Tarif A., Carpenter S., Müller-Doblies U. (2007). Clinical signs and pathology shown by British sheep and cattle infected with bluetongue virus serotype 8 derived from the 2006 outbreak in northern Europe. Vet. Rec..

[69] Souza A.H., Silva E.P., Cunha A.P., Gümen A., Ayres H., Brusveen D.J., Guenther J.N., Wiltbank M.C. (2011). Ultrasonographic evaluation of endometrial thickness near timed AI as a predictor of fertility in high-producing dairy cows. Theriogenology.

[70] De Smit A.J., Bouma A., Terpstra C., van Oirschot J.T. (1999). Transmission of classical swine fever virus by artificial insemination. Vet. Microbiol..

[71] World Organisation for Animal Health (OIE) (2020). Chapter 4.7: Collection and Processing of Bovine, Small Ruminant and Porcine Semen. https://www.oie.int/index.php?id=169&L=0&htmfile=chapitre_coll_semen.htm.

[72] European Commission, DG Health and Consumers, Animal Health and Welfare Council Directive 2000/75/EC of 20 November 2000 Laying Down Specific Provisions for the Control and Eradication of Bluetongue. https://eur-lex.europa.eu/legal-content/EN/TXT/PDF/?uri=CELEX:32000L0075&from=EN.

[73] European Union Regulation (EU) 2016/429 of the European Parliament and of the Council of 9 March 2016 on Transmissible Animal Diseases and Amending and Repealing Certain Acts in the Area of Animal Health (‘Animal Health Law’). https://eur-lex.europa.eu/legal-content/EN/TXT/PDF/?uri=CELEX:32016R0429&from=NL.

[74] European Commission, DG Health and Consumers, Animal Health and Welfare Bluetongue, Overview Seasonally Vector Free Periods 2009–2010. https://ec.europa.eu/food/sites/food/files/animals/docs/ad_control-measures_bt_overview_seasonally_vfp_2009-2010.pdf.

[75] Carpenter S., Wilson A., Barber J., Veronesi E., Mellor P., Venter G., Gubbins S. (2011). Temperature dependence of the extrinsic incubation period of orbiviruses in Culicoides biting midges. PLoS ONE.

